# The burden of non-communicable diseases attributable to high BMI in Brazil, 1990–2017: findings from the Global Burden of Disease Study

**DOI:** 10.1186/s12963-020-00219-y

**Published:** 2020-09-30

**Authors:** Mariana Santos Felisbino-Mendes, Ewerton Cousin, Deborah Carvalho Malta, Ísis Eloah Machado, Antonio Luiz Pinho Ribeiro, Bruce Bartholow Duncan, Maria Inês Schmidt, Diego Augusto Santos Silva, Scott Glenn, Ashkan Afshin, Gustavo Velasquez-Melendez

**Affiliations:** 1 Nursing School, Department of Maternal and Child Nursing and Public Health, Universidade Federal de Minas Gerais, Belo Horizonte, Brazil; 2 Postgraduate Program in Nursing, Nursing School, Universidade Federal de Minas Gerais, Belo Horizonte, Brazil; 3 Postgraduate Program in Epidemiology, Universidade Federal do Rio Grande do Sul, Porto Alegre, Brazil; 4 Institute for Health Metrics and Evaluation, University of Washington, Seattle, USA; 5 School of Medicine, Department of Family Medicine, Mental and Public Health, Universidade Federal de Ouro Preto, Ouro Preto, Brazil; 6 School of Medicine, Department of Internal Medicine and Hospital das Clínicas, Telehealth Center, Universidade Federal de Minas Gerais, Belo Horizonte, Brazil; 7 Departamento de Educação Física, Universidade Federal de Santa Catarina, Florianópolis, Brazil

**Keywords:** Obesity, Body mass index, Risk factors, Comparative risk assessment, Cardiovascular disease

## Abstract

**Background:**

The prevalence and burden of disease resulting from obesity have increased worldwide. In Brazil, more than half of the population is now overweight. However, the impact of this growing risk factor on disease burden remains inexact. Using the 2017 Global Burden of Disease (GBD) results, this study sought to estimate mortality and disability-adjusted life years (DALYs) lost to non-communicable diseases caused by high body mass index (BMI) in both sexes and across age categories. This study also aimed to describe the prevalence of overweight and obesity throughout the states of Brazil.

**Methods:**

Age-standardized prevalence of overweight and obesity were estimated between 1990 and 2017. A comparative risk assessment was applied to estimate DALYs and deaths for non-communicable diseases and for all causes linked to high BMI.

**Results:**

The prevalence of overweight and obesity increased during the period of analysis. Overall, age-standardized prevalence of obesity in Brazil was higher in females (29.8%) than in males (24.6%) in 2017; however, since 1990, males have presented greater rise in obesity (244.1%) than females (165.7%). Increases in prevalence burden were greatest in states from the North and Northeast regions of Brazil. Overall, burden due to high BMI also increased from 1990 to 2017. In 2017, high BMI was responsible for 12.3% (8.8–16.1%) of all deaths and 8.4% (6.3–10.7%) of total DALYs lost to non-communicable diseases, up from 7.2% (4.1–10.8%), and 4.6% (2.4-6.0%) in 1990, respectively. Change due to risk exposure is the leading contributor to the growth of BMI burden in Brazil. In 2017, high BMI was responsible for 165,954 deaths and 5,095,125 DALYs. Cardiovascular disease and diabetes have proven to be the most prevalent causes of deaths, along with DALYs caused by high BMI, regardless of sex or state.

**Conclusions:**

This study demonstrates increasing age-standardized prevalence of obesity in all Brazilian states. High BMI plays an important role in disease burdens in terms of cardiovascular diseases, diabetes, and all causes of mortality. Assessing levels and trends in exposures to high BMI and the resulting disease burden highlights the current priority for primary prevention and public health action initiatives focused on obesity.

## Background

The overall disease prevalence and burden related to obesity have increased worldwide. The prevalence of obesity showed a continuous increase between 1980 and 2015 and doubled in 73 countries during this same period [1, 2]. This increase is more often observed in low- and middle-income countries, and, in some nations, it is more prominent among women [3].

A high body mass index (BMI) is one of the major causes of several diseases worldwide, constituting an important risk factor for cardiovascular diseases (CVDs) [4], type 2 diabetes mellitus [4], and neoplasms [5], among others. It is also associated with many musculoskeletal disorders, such as arthritis [6]. Globally, four million deaths and the loss of 120 million disability-adjusted life years (DALYs) were attributable to high BMI in 2015 [2]. Moreover, BMI has proven to be a feasible indicator of adiposity [7] and is widely used in epidemiological studies, especially those which assess time trends in various populations and subgroups.

In Brazil, more than half of the population is overweight [8, 9]. The prevalence of obesity was generally higher among women and has been rising quickly in risk factor rankings [10]. However, the impact of the rise in BMI causing disease burdens remains unknown and should be more specifically assessed in order to better address this problem. To the best of our knowledge, no prior studies have estimated the potential effect of weight gain outcomes in Brazil and their impacts on those diseases, nor have they analyzed well-known conditions associated with an excess of weight, such as non-communicable diseases (NCDs).

In parallel, there is an important gap in national or subnational estimates, which would allow one to better evaluate public policies concerning health promotion and healthy weight management. Disease burden estimates attributable to a given risk factor could be assessed by using a model design of comparative risk assessment [11]. This model allows one to quantify the number and proportions of deaths and DALYs lost due to non-communicable disease, which would have been prevented if the risk factor—in this specific case of high BMI—had been maintained at ideal levels [12].

Considering this scenario, using the 2017 national and subnational Global Burden of Disease (GBD) study results, the present study aimed to estimate the number and proportion of deaths and DALYs of non-communicable diseases caused by high BMI in both sexes and across age categories. This study also aimed to describe trends in the prevalence of overweight and obesity throughout the states of Brazil.

## Methods

### Global Burden of Disease (GBD) estimates

This study used estimates from the Institute for Health Metrics and Evaluation (IHME) for Brazil, as reported in the 2017 GBD study. Brazilian data used by IHME were provided by the Brazilian Ministry of Health and the Brazilian Institute of Geography and Statistics (IBGE). Estimates are available to the public and can be accessed at http://vizhub.healthdata.org/gbd-compare.

The Global Burden of Diseases, Injuries, and Risk Factors study provides the most recent epidemiological data according to year, age, and sex from 195 countries and territories, including Brazil [13]. Annually, it formulates an up-to-date comprehensive assessment of the evidence for national and subnational risk factor exposures and the main causes of the disease burden [13, 14].

Data sources resulted from a systematic analysis of published studies and available data sources. BMI data sources were gathered from multi-country survey programs, national surveys, and longitudinal studies, which were available in the Global Health Data Exchange (GHDx), which can be accessed at http://ghdx.healthdata.org/. In Brazil, BMI data were extracted from published papers, the National Health Survey [15], the Consumer Expenditure Survey (POF) [16], the Telephone Survey Surveillance System for Risk and Protective Factors for Chronic Diseases (Vigitel) [8], local surveys, and birth cohorts.

After adjusting for self-report bias and splitting aggregated data into 5-year age-sex groups, the spatiotemporal Gaussian process regression (ST-GPR) was used to estimate the prevalence of overweight and obesity [13, 17]. This modeling approach has been previously described in detail [17].

### Definition of High BMI

The level of exposure that is associated with the lowest risk is called the theoretical minimum risk exposure level (TMREL). The TMREL is used to calculate what could be prevented in the burden of disease if, in the past, the population exposure would have been sustained at a theoretical minimum risk of exposure [10, 13].

The TMREL of BMI was determined based on the BMI level, which was associated with the lowest risk of all-cause mortality in prospective cohort studies. From the 2015 GBD on, based on the findings of the most recently pooled analysis of prospective cohorts [18], a BMI change in the TMREL, from 21–23 to 20–25 kg/m^2^, occurred in adults, a cutoff that remained in the 2017 estimates [13, 14, 17].

### Population attributable fraction (PAF)

The population attributable fraction (PAF) estimates the reduction of a determined health metric, such as the number and proportions of deaths or DALYs in a specific case scenario, where the optimal level of exposure (TMREL) would be compared to the altered level of the same risk factor, through the PAF formula [19, 20] for a continuous risk factor, as explained elsewhere [17]. Thus, the formula includes the relative risk per 5-unit change in BMI for each disease endpoint. In this case, the range of 20–25 km/m^2^ was assumed to be optimal when the mortality or morbidity risks were the lowest in the adult population [17, 18].

Moreover, GBD methodology includes four key components in the estimation of the burden attributable to a given risk factor: (1) the metric of burden being assessed (the number of deaths, years of life lost [YLLs], years lived with disability [YLDs], or DALYs [the sum of YLLs and YLDs]); (2) the exposure levels for a risk factor; (3) the relative risk of a given outcome due to exposure; and (4) the counterfactual level of risk factor exposure [13, 14]. All modeling is performed using a Bayesian meta-regression model (DisMod-MR 2.1) [13, 17].

### Data analysis

This study first analyzed estimates of age-standardized prevalence and its 95% uncertainty interval (95%UI) of overweight (BMI ≥ 25.0 kg/m^2^) and obesity (BMI ≥ 30 kg/m^2^) for the adult population of 20 years old and older between 1990 and 2017, in both sexes and across states. The percent change in prevalence from 1990 to 2017, with its uncertainty intervals, was then assessed.

Second, the portion of death and DALY rates that could be attributable to high BMI in 2017 for all causes, and mostly for non-communicable diseases, including CVDs, diabetes, chronic kidney diseases, and neoplasms, was analyzed. All estimates were extracted according to age, sex, and states.

Third, this study analyzed the trends of mortality and DALY rates attributable to high BMI in the period from 1990 to 2017, for men and women. Also, evaluated were the decomposition of the percent changes (1990–2017) in death and DALY rates attributed to the high BMI found in four factors: (1) population growth, (2) population age structure, (3) risk-deleted DALY rates, and 4) high BMI exposure [13, 17, 21]. In summary, risk-deleted rates are death and DALY rates that would have been observed if the high BMI risk factor had been reduced to the TMREL levels, multiplied by one minus PAF. The methodology adopted by the GBD study also produces linear risk-aggregates over time and is described elsewhere in more detail [13, 17, 21].

The GBD’s world population age standard has been described elsewhere [22]. The GBD Brazil study was approved by the Research Ethics Committee of Universidade Federal de Minas Gerais (UFMG) (CAAE—62803316.7.0000.5149).

## Results

Table 1 shows adult age-standardized prevalence for the adult population of 20 years old and older, with 95% uncertainty intervals (UI) of overweight (BMI ≥ 25.0Kg/m^2^) in Brazil and its federal units, determined according to sex. In 2017, overall prevalence was 54.3% (95% UI 52.0–56.6) for men and 52.4% (95% UI 49.8–55.0) for women, showing no statistically significant difference by sex. More than half of the population was overweight in Brazil in most states, except for Maranhão and Piauí. The states with prevalence above the national rate were, in decreasing order of importance, Distrito Federal (73.8 and 70.4%), Mato Grosso do Sul (58.4 and 54.5%), Rio Grande do Sul (57.8 and 54.1%), Mato Grosso (57.2 and 53.9%), Paraná (56.4 and 52.7%), São Paulo (56.2 and 53.4%), Acre (55.6 and 55.8%), Rio de Janeiro (55.6 and 52.4%), Roraima (54.8 and 54.0%), Amazonas (54.7 and 57.0%), and Ceará (54.6 and 53.4%) for both men and women, respectively; Amapá (56.3%), Rondônia (53.2%), Rio Grande do Norte (52.9%), and Pernambuco (52.5%) for women only, and Santa Catarina (56.2%) for men only. This table also shows the percent change in prevalence in the period from 1990 to 2017. A similar increase for men, 47.6% (95%UI 36.4–59.8), and women, 47.6% (95%UI 34.6–61.3), as observed. This increase was above 50% in most of the federated units and above 70% in most of the Northern and Northeastern states, and was most commonly found in women.

**Table 1:** Overweight (BMI ≥ 25.0 Kg/m^2^) and obesity (BMI ≥ 30.0 kg/m^2^) adult age-standardized prevalence in 2017 and percent change (APC) and 95% uncertainty intervals from 1990 to 2017 for Brazil and 27 states

Location	Sex	Age-standardized overweight prevalence, 2017		Percent change in overweight prevalence, 1990–2017		Age-standardized obesity prevalence, 2017		Percent change in obesity prevalence, 1990–2017	
		%	95% UI	%	95% UI	%	95% UI	%	95% UI
Brazil	Male	54.3	52.0–56.6	47.6	36.4 -59.8	24.6	22.8–26.5	244.1	196.3–302.3
	Female	52.4	49.8–55.0	47.6	34.6–61.3	29.8	27.9–31.8	165.7	133.8–202.6
Acre	Male	55.6	52.4–58.8	76.8	54.4–103.2	24.8	22.4–27.5	338.2	246.4–450.0
	Female	55.8	52.1–59.5	74.2	48.8–102.1	32.0	28.9–35.0	251.9	187.7–331.8
Alagoas	Male	51.9	48.8–55.2	85.6	62.5–113.3	21.2	19.0–23.7	354.4	258.8–491.2
	Female	50.6	47.1–54.1	87.1	61.6–118.9	28.0	25.6–30.9	270.3	207.6–362.1
Amazonas	Male	54.7	51.5–57.7	51.5	34.0–71.7	26.8	24.1–29.8	274.8	197.4–370.6
	Female	57.0	53.1–60.7	67.5	44.6–95.2	32.4	29.4–35.4	200.5	148.4–264.7
Amapá	Male	54.2	50.9–57.4	71.2	49.9–95.6	25.9	23.1–28.7	315.8	228.7–423.5
	Female	56.3	52.5–60.0	81.9	54.9–111.9	32.2	29.3–35.3	255.5	191.1–338.2
Bahia	Male	47.8	44.9–50.8	64.3	45.2-–89.1	18.5	16.6–20.5	277.8	200.2–374.3
	Female	51.4	47.7–54.7	70.3	46.6–97.7	27.8	25.3–30.5	217.0	157.9–290.4
Ceará	Male	54.6	51.4–58.0	70.0	49.9–92.5	24.9	22.3–27.5	310.4	227.0–415.8
	Female	53.4	49.8–57.1	79.0	54.8–108.8	29.4	26.8–32.1	247.7	184.1–329.0
Distrito Federal	Male	73.8	69.5–78.0	27.1	13.3–42.5	48.0	43.4–52.7	185.3	130.8–255.0
	Female	70.4	65.8–75.5	31.9	15.7–50.3	51.9	47.4–57.1	126.4	89.3–170.7
Espírito Santo	Male	53.9	50.8–57.1	46.0	27.8–67.4	23.4	21.1–26.0	229.8	165.2–311.1
	Female	51.7	48.1–55.3	59.2	37.3–85.8	29.0	26.1–31.8	187.6	135.9–250.9
Goiás	Male	52.9	50.0–56.0	58.7	39.0–80.8	21.4	19.3–23.8	271.0	195.3–367.1
	Female	51.1	47.7–54.7	60.3	39.2–86.6	28.4	25.8–31.0	205.1	149.6–271.6
Maranhão	Male	47.1	44.2–50.3	88.3	64.2–119.0	16.9	15.1–18.9	372.2	266.8–518.4
	Female	47.2	43.8–50.6	96.6	67.6–130.3	23.8	21.6–26.0	297.2	220.5–396.1
Minas Gerais	Male	51.5	48.5–54.5	54.6	36.4–74.8	21.3	19.3–23.4	243.3	179.0–320.9
	Female	50.0	46.8–53.5	23.5	8.7–42.1	27.0	24.7–29.4	112.1	77.7–156.4
Mato Grosso do Sul	Male	58.4	54.9–61.6	66.8	46.6–89.2	27.0	24.4–29.8	301.1	222.5–400.8
	Female	54.5	51.0–58.1	71.0	48.5–96.4	31.7	29.1–34.6	235.6	176.8–310.0
Mato Grosso	Male	57.2	54.1–60.7	52.7	35.6–72.8	25.6	23.1–28.5	242.3	174.0–327.9
	Female	53.9	50.4–57.5	53.1	32.7–77.2	30.5	27.6–33.5	182.6	134.5–247.2
Pará	Male	53.6	50.6–56.7	59.4	40.6–83.5	22.7	20.2–25.1	248.3	174.4–338.5
	Female	50.7	47.3–54.0	75.7	51.4–106.4	26.1	23.8–28.7	219.7	158.9–294.2
Paraíba	Male	52.6	49.5–55.8	50.6	32.8–70.4	22.9	20.5–25.5	231.2	164.1–318.1
	Female	50.6	47.2–53.9	54.4	33.1–78.1	27.0	24.5–29.7	178.8	128.8–239.2
Paraná	Male	56.4	53.3–59.8	50.6	34.0–72.2	24.7	22.3–27.3	253.9	184.1–343.6
	Female	52.7	49.5–56.3	51.9	32.1–74.4	29.1	26.7–31.7	172.7	128.9–230.7
Pernambuco	Male	52.1	49.4–55.2	55.3	37.7–78.2	22.1	20.0 –24.6	258.2	191.2–353.0
	Female	52.5	49.2–56.0	61.1	39.5–85.2	27.5	25.1–30.0	191.8	141.3–252.6
Piauí	Male	47.7	44.6–50.8	76.8	54.6–105.0	18.6	16.5 –20.9	313.9	227.9–432.7
	Female	46.8	43.5–50.4	79.9	55.1–109.9	23.9	21.6–26.4	245.4	180.5–333.1
Rio de Janeiro	Male	55.6	52.2–58.7	32.9	17.8–49.5	28.4	25.6 –31.5	225.1	164.1–302.7
	Female	52.4	48.9–55.6	33.8	17.2–54.1	32.2	29.6–35.0	141.3	101.3–188.6
Rio Grande do Norte	Male	53.5	50.4–56.7	64.9	43.1–90.8	24.4	21.8–27.1	292.3	206.6–398.1
	Female	52.9	49.3–56.8	75.4	52.4–105.6	28.3	25.8–31.1	238.6	177.6–318.0
Rondônia	Male	53.8	50.2–56.9	68.5	47.6–92.6	24.1	21.7–26.8	293.6	211.6–397.3
	Female	53.2	49.8–56.9	69.6	46.9–96.2	30.1	27.2–33.0	216.0	158.9–287.8
Roraima	Male	54.8	51.8–58.0	64.5	43.7–88.7	23.7	21.2–26.6	281.9	201.2–383.5
	Female	54.0	50.3–57.7	71.8	48.0–98.9	30.1	27.5–32.8	222.0	166.5–291.4
Rio Grande do Sul	Male	57.8	54.7–60.8	38.5	23.0–56.5	25.5	23.1–28.1	207.1	149.9–278.4
	Female	54.1	50.8–57.6	43.1	24.7–63.1	32.5	29.8–35.7	150.4	109.1–201.8
Santa Catarina	Male	56.2	52.9–59.7	38.3	22.6–56.2	25.3	22.7–27.9	227.7	162.3–313.2
	Female	50.4	47.4–54.0	45.9	27.7–67.3	28.3	25.9–31.1	152.2	108.0–208.0
Sergipe	Male	52.9	49.6–56.3	66.8	46.5–91.1	24.0	21.6–26.7	305.0	224.5–409.2
	Female	51.1	47.7–54.5	65.9	42.9–92.7	28.5	25.9–31.2	208.6	151.1–283.8
São Paulo	Male	56.2	53.3–59.1	35.3	22.3–50.4	27.7	25.1–30.4	231.1	173.5–298.7
	Female	53.4	50.0–56.6	36.9	21.4–54.5	31.8	29.2–34.4	149.3	112.3–192.8
Tocantins	Male	51.9	49.0–54.8	74.6	52.0–100.9	20.4	18.1–22.7	311.1	222.2–422.3
	Female	49.2	45.9–52.6	83.8	57.0–117.0	26.5	24.1–29.1	259.5	191.9–342.8

*95% UI* 95% uncertainty intervals, *BMI* body mass index

This same table shows the adult age-standardized prevalence of obesity (BMI ≥ 30.0 kg/m^2^), which was higher among women, 29.8% (95%UI 27.9–31.8), than among men, 24.6% (95%UI 22.8–26.5). An increased prevalence for both sexes was observed in Acre, Amazonas, Amapá, Distrito Federal, Mato Grosso do Sul, Mato Grosso, Rio Grande do Sul, Rio de Janeiro, and São Paulo. By contrast, an increased prevalence only among women was found in Rondônia and Roraima, as compared to Paraná, Ceará, and Santa Catarina where the prevalence was found mostly among men. The percent change in obesity prevalence from 1990 to 2017 was 244.1 (95% UI 196.3–302.3) for men and 165.7 (95% UI 133.8–202.6) for women. An annual increase of over 300% was observed in most of the North and Northeast states for men. Trends of overweight and obesity during this same period were accessed and are presented in an additional file (supplementary Figure 1).

Regarding the disease burden, overall, in 2017, high BMI was responsible for an estimated 12.3% of total deaths and 8.4% of total DALYs for all causes. Thus, this risk factor was responsible for 165,954 deaths and 5,095,125 DALYs. High BMI attributable deaths and DALYs were more important in women (14.6%, 95% UI 10.7–18.9 and 9.4%, 95% UI 7.2–11.7, respectively) than in men (10.5%, 95% UI 7.2–14.1 and 7.7%, 95% UI 5.5–10.0, respectively). Specifically, 16.2% (95% UI 11.6–221.1) of deaths and 11.9% (95%UI 8.8–15.1) of DALYs due to non-communicable diseases were attributable to high BMIs, but no statistically significant differences were observed between the sexes.

**Fig. 1:**
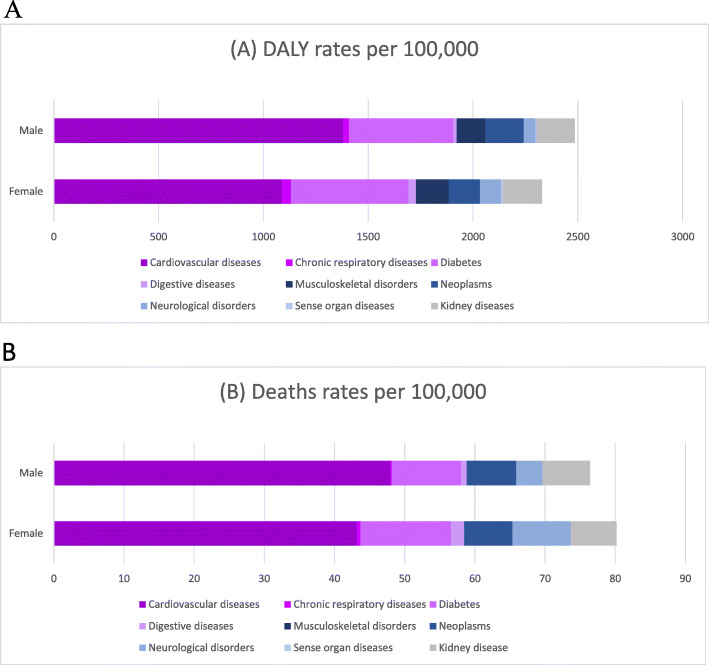
Death (**a**) and DALY (**b**) rates per 100,000 attributable to High BMI by sex, Brazil, 2017. Legend: Deaths (**a**) and DALY (**b**) rates per 100,000 of non-communicable diseases related to high BMI are shown for males and females. Numbers in the x-axis represent the rates. DALYs, disability-adjusted life-years; BMI Body mass index

CVDs and diabetes, followed by neoplasms, chronic kidney diseases, and neurological disorders, for both sexes, were the leading causes through which high BMI acts to cause disease burdens (Fig. 1). This same graph shows differences between the sexes, such as death rates, which were more prominent among women, particularly for diabetes and neurological disorders, and DALYs, which were higher among men, including CVDs and neoplasms. However, when considering the uncertainty intervals, no statistically significant difference was observed (supplementary Table 1). The same occurred among women, as high BMI also proved to be more associated with DALY rates of musculoskeletal disorders, whereas the UIs overlapped.

Approximately, 96,347 deaths occurred due to CVDs resulting from high BMIs in 2017, which represents 7.1% of all deaths, as compared to 2,605,648 DALYs, representing 4.3% of all DALYs.

**Fig. 2:**
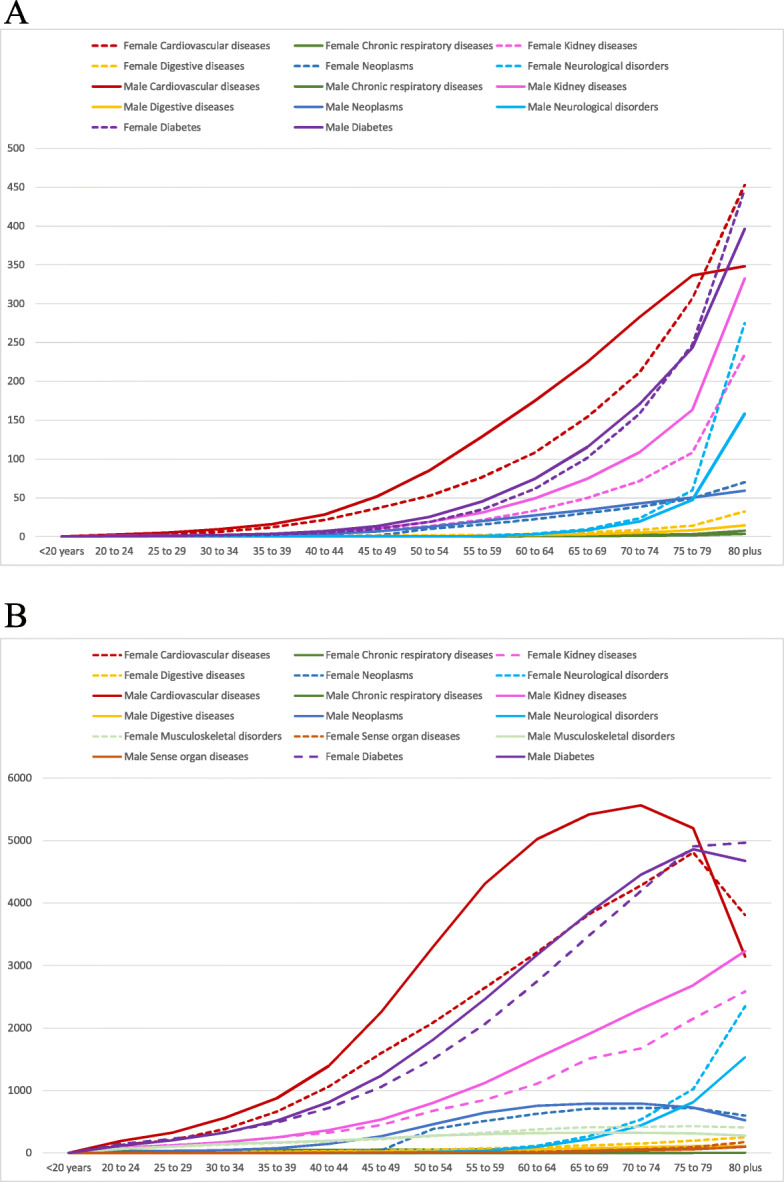
Death (**a**) and DALY (**b**) rates per 100,000 of specific causes attributable to high BMI according to age and by sex. Brazil, 2017. Legend: Death (**a**) and DALY (**b**) rates per 100,000 related to high BMI are shown for females (dotted line) and males (full line) and across age categories. Each line is a specific cause attributable to high BMI. The y-axis has the death (**a**) and DALY (**b**) rates and the x-axis the age categories. DALYs, disability-adjusted life-years; BMI, body mass index

Figure 2 shows death and DALY rates related to BMI in both sexes and across age categories. Death rates for diabetes and CVDs caused by high BMI begin to increase after 40 years of age, whereas neoplasms begin to increase after 50 years of age and neurological disorders after 60 years of age, for both sexes. As for DALYs, an early onset of these burdens was noticed at around 30 years of age due to CVDs, diabetes, and urogenital, blood, and endocrine diseases, and at around 40 years of age for musculoskeletal diseases, for both sexes. Regarding neoplasms, a statistically significant difference was observed between the sexes, which increased considerably after 35 years of age among men and after 50 years of age among women.

**Fig. 3:**
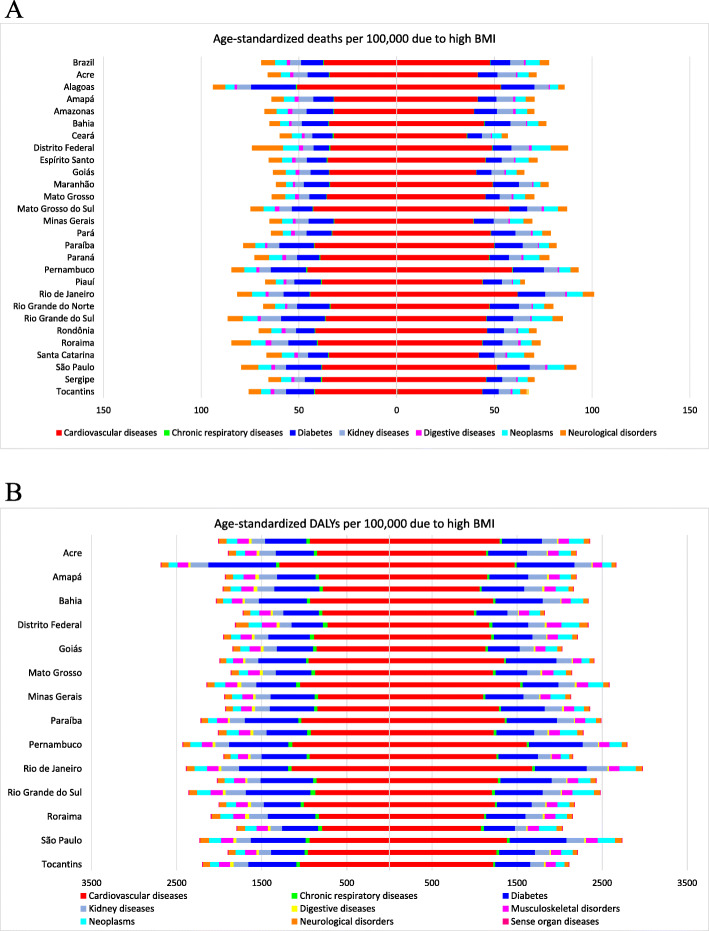
Age-standardized Death (**a**) and DALY (**b**) rates per 100,000 attributable to high BMI across all states by sex, women on the left and men on the right. Legend: Colored bars are the attributable age-standardized DALY and death rates corresponding to high BMI, each color a specific cause, across all Brazilian states, 2017. Women are on the left and men on the right. DALYs, disability-adjusted life-years; BMI, body mass index

Moreover, Fig. 3 shows DALY and death rates caused by high BMI across all Brazilian states according to sex in 2017. Some states, such as Alagoas, Pernambuco, and Rio de Janeiro, present a higher burden attributable to high BMIs than the average reported in Brazil, with higher death rates due to CVDs and diabetes mellitus. A higher death rate due to neurological disorders was observed in the Federal District of Brasília, more deaths due to neoplasms among women in Roraima, and higher death rates due to CVDs among men in Mato Grosso do Sul, all attributable to high BMI. It was observed that these same states have higher rates of DALYs attributable to high BMIs, although differences are non-significant when considering the 95% UI (supplementary Table 1) (Fig. 3b).

**Fig. 4:**
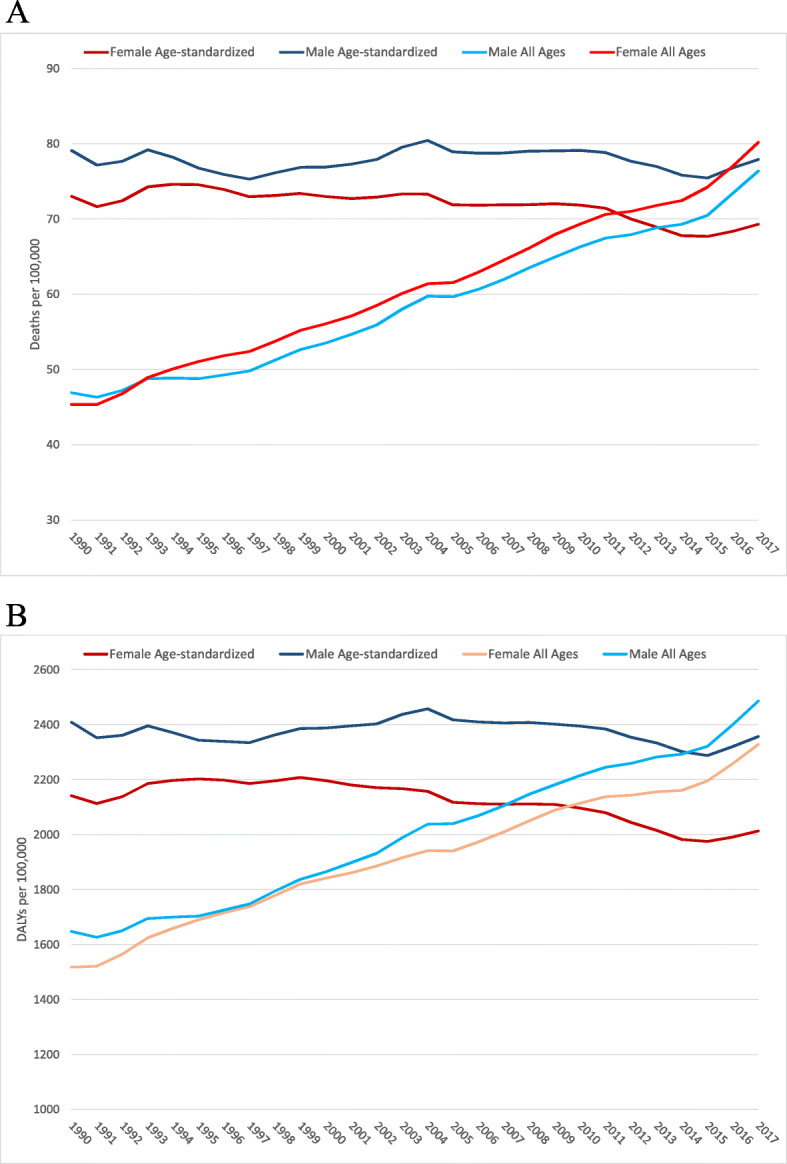
Trends of mortality (**a**) and DALY (**b**) rates, all ages and age-standardized attributable to high BMI by sex, 1990-2017, Brazil. Legend: Crude and age-standardized deaths and DALY rates attributable to high BMI, by sex, are shown. Y-axis presents the rates and x-axis the years. The line shows the trends in the period of 1990–2017. DALYs, disability-adjusted life-years; BMI, body mass index

Figure 4 shows that the crude death rates attributable to high BMI increased 61.4% and 57%, for men and women, respectively, between 1990 and 2017, from 46.9 (UI 95% 24.8–72.7) and 45.4 (27.5–65.7) deaths per 100,000 in 1990 to 76.4 (UI 95% 52.1–103.1) and 80.2 (58.7–103.3) deaths per 100,000 in 2017. In this same period, the crude DALY rates rose 51% and 53%, for men and women, respectively, from 1647.6 (UI 95% 904.6–2477.6) and 1517.2 (95% UI 956.6–2157.2) per 100,000 in 1990 to 2485.7 (95% UI 1772.6–3259.5) and 2328.8 (95% UI 1770.4–2926.7) per 100,000 in 2017. When these rates were age-standardized, relatively stable curves are observed.

**Fig. 5:**
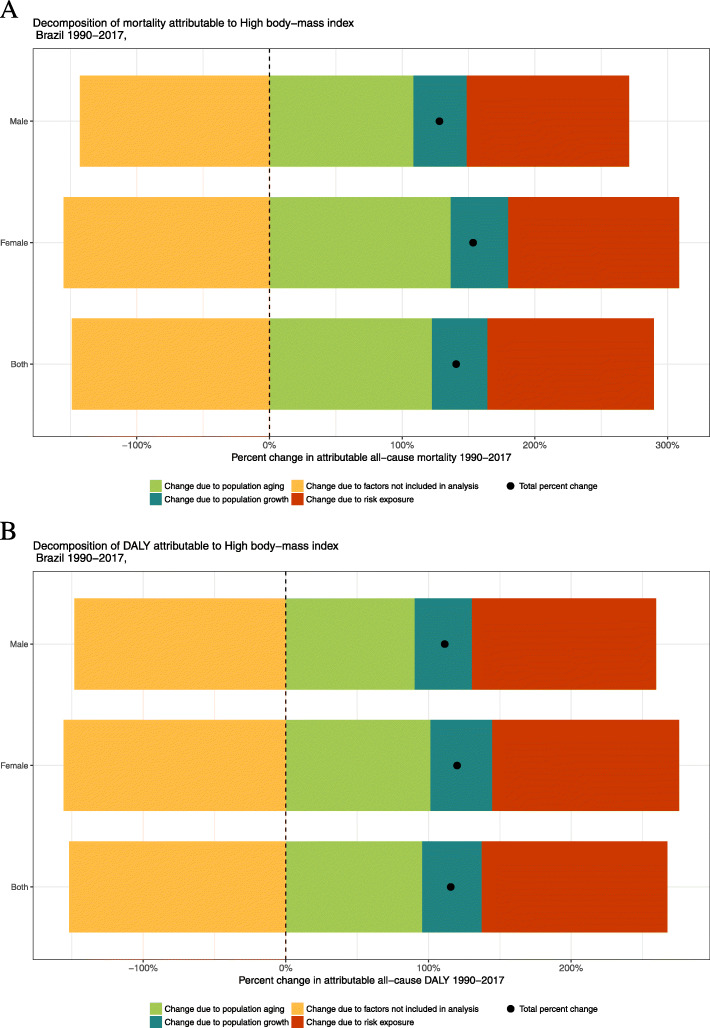
Decomposition of deaths (**a**) and DALYs (**b**) attributable to high BMI, Brazil 1990-2017. Legend: Percent change in risk-attributable deaths and DALYs. Results are shown for all causes combined. The black dot shows total percentage change. The risk-deleted DALY rate is the expected DALY rate if the exposure level for high BMI was reduced to the theoretical minimum risk exposure level. DALYs, disability-adjusted life-years; BMI, body mass index

At last, Fig. 5 shows decomposition of percent changes in all-cause deaths and DALY numbers related to high BMIs from 1990 to 2017. For deaths, the change due to population aging was responsible for 122% of the increase, while the change in risk exposure accounted for 125% and population growth was responsible for 42%, and the change in risk-deleted rates was responsible for a decrease of 149%. The change due to population aging contributed to an increase of 137% and 109% for women and men, respectively, an increase of 26% in females. In terms of DALYs, for both sexes, population aging accounted for an increase of 96%, changes in risk exposure increased by 130%, while 42% was due to population growth. On the other hand, the change in risk-deleted rates decreased by 152%.

## Discussion

The present study demonstrates an increasing age-standardized prevalence of overweight and obesity in the Brazilian adult population and states from 1990 to 2017. The overall age-standardized prevalence of obesity in Brazil was higher in females than males in 2017, but males presented a higher increase than did females during the same period. The percent change was higher in the Northern and Northeastern states, which, for the most part, are less developed and lower income regions.

This increase has been observed in previous national surveys [8, 23–26] and among children and adolescents [27]. Similar results were found when comparing countries of different income levels, where there was an increase in people’s excess of weight in low- and middle-income countries (LMIC), while lower levels were observed in rich nations [3, 28]. This implies the persistence of malnutrition, but now in the form of overnutrition rather than undernutrition [3, 25].

Drivers of this phenomenon have been described in detail in previous studies, such as changes in the food environment, greater supply of high energy foods, marketing, urbanization, and the built environment, which have also cut down time and space for physical activities [2, 29–31]. Moreover, as a metabolic condition, high BMI is an intermediary risk factor in the causal model of NCDs, which is usually a consequence of other important risk factors, such as inadequate diet and physical inactivity, which have also proven to have reached levels in Brazil that are even higher than those of high BMIs [10, 32].

Therefore, high BMI exposure is increasing and driving up its attributable burden throughout Brazil, corroborating global findings for this specific risk factor [2, 13, 14, 17]. Our study shows that high BMI played an important role in the national burden (deaths and DALYs) of NCDs, especially those related to CVDs and diabetes mellitus.

Prior studies have shown a relationship between excess weight and mortality for all causes in the four continents [18]. The present study has more specifically shown the burden of NCDs due to this condition, as well as estimates of DALYs due to excess weight. The present study results are similar to those found for the Eastern Mediterranean countries [33]. Furthermore, more than 60% of overweight individuals live in LMICs, and, in these settings, the relationship of high BMI and morbidity has also been understudied [34].

The attributable fraction of NCD burden due to high BMI revealed no sex-specific pattern. Although death rates were more prominent among women, particularly for diabetes mellitus and neurological disorders, and DALYs were higher among men when considering CVD and neoplasms, uncertainty intervals overlapped. Nevertheless, it is important to note that NCDs have proven to be responsible for the greatest burden of death and disability among women, highlighting CVD, diabetes mellitus, dementia, depression, and musculoskeletal disorders [35]. As such, the prevention of NCDs should be more advocated for women, as the healthcare agenda for women commonly gives priority to sexual and reproductive health issues. Our study results indicate that an important part of this burden is due to high BMIs. Moreover, a recent study has also shown a greater increase in obesity prevalence among Brazilian women of childbearing age [23], which are relatively young women. Moreover, a considerable number of premature deaths have been observed due to CVDs caused by high BMIs among men of young ages, which might contribute to higher DALY rates, as observed through the YLL component of this measurement.

Mechanisms underlying the consequences of obesity leading to NCDs have also been explained in previous reports, especially regarding CVD and DM [36–38]. High BMI mostly causes a chronic systemic inflammation and higher sympathetic activity, which can contribute to insulin resistance and hypertension, respectively [37], leading to endothelial dysfunction and atherosclerosis [38]. Thus, its effect is primarily mediated through other intermediary risk factors, such as hypertension, hypercholesterolemia, and hyperglycemia, the last two also known as metabolic risk factors [37]. Recent evidence also highlights the relation between obesity and neurological disorders in the hippocampal structure and function [39]. The present study observed a higher burden of neurological disorders due to high BMIs among individuals of 60 years old and older.

The overall burden due to high BMI increased from 1990 to 2017. When considering the decomposition of percent changes in all-cause DALYs and deaths related to this specific risk in the studied period, high BMI trends in attributable mortality and DALYS are driven by the interplay between population aging, an increase in risk exposure, and population growth. Moreover, change due to risk exposure is the leading contributor to growth in overweight and obesity burden in Brazil, followed by population aging, rather than the population growth, which had a lesser contribution. This understanding of drivers is crucial to subsidize public policies and interventions.

To the best of our knowledge, these are the first national and subnational estimates of these two metrics of the burden of NCDs attributable to high BMIs in Brazil. The increased in NCDs in Brazil is a great challenge, and to tackle it, its main risk factors, such as high BMI, must be addressed. The subnational approach is an important advance, as it allows for the evaluation of important disparities within the country and helps in the creation of local policies adjusted to the reality of each setting.

Thus, the present study advances this matter, and its findings are crucial for the development of an approach to public health, including the renewal of surveillance, management, and prevention policies in Brazil. Public policies, like the “Strategic Actions Plan for Coping with NCDs in Brazil, 2011-2022” [40], have been of great use in guiding surveillance at the national level of NDCs and their risk factors, such as high BMI, but further advances through interventions are required.

Since 2011, various polices have been established to address this problems in Brazil [39, 41, 42], but no major population success has yet been shown, corroborating a scenario found for other nations as well [2]. Thus, despite of this great challenge, it represents an opportunity to tackle disease burdens, especially considering the fact that developed countries have made some progress in facing overweight [3].

Moreover, promoting healthier individual lifestyles, focused on diet and physical activity strategies and interventions, is necessary, especially in Northern and Northeastern states, but it is not enough. New approaches should consider measures that take into account the patient’s entire lifespan [43], such as intergenerational cycles and focus on childhood [44].

Healthcare providers should be more aware of simple measures in primary health care. Height and weight can be easily accessed, followed by a simple calculation of BMI, which could detect an important risk factor for greater health burdens, including death. On the other hand, weight loss could act directly in the reduction of incidences of many diseases, such as diabetes mellitus [45] and CVD [46].

This study has limitations, which should be considered. First, GBD used both self-reported and measured data with respect to height and weight. On the other hand, GBD methodology corrected the bias in self-reported data, using measured data for each age, sex, and geographic region, as previously described [17]. High agreement between measured and self-reported anthropometric data was demonstrated, using several statistical methods across sex, age group, education level, and household location categories, which reinforces their use as proxies of measured values in the Brazilian population ≥ 18 years old [47]. Moreover, GBD methodology and estimates have been used worldwide and approved as valid in several countries.

Estimates before 2000 should be more carefully considered, as national surveys monitoring this risk factor were less frequently applied. Furthermore, a possible impact of pre-existing diseases and other confounding factors, such as smoking and other lifestyle habits, cannot be excluded, which could not be evaluated, but are usually excluded from the observational studies, which are sources of relative risk estimation [2]. Moreover, this limitation should be taken cautiously, as there is a well-established biological mechanism between adiposity tissue accumulation and metabolic diseases, such as diabetes [36].

## Conclusions

Prevalence of overweight and obesity continues to rise in the Brazilian population, with some variability across states and regions. Additionally, the burden of NCDs associated with high BMI should be considered high, especially in times when abundant evidence highlighting obesity as a major risk factor for NCDs is available. These results signal the need for increased attention to healthcare programs aimed at maintaining healthy weight in order to tackle the NCD burden in Brazil.

## Supplementary information

**Additional file 1: Figure S1.** Prevalence trends of overweight (BMI ≥ 25.0 Kg/m2) (A) and obesity (BMI ≥ 30.0 kg/m2) (B) during the period of 1990 and 2017 in Brazil for both sexes (green), females (orange) and males (blue). The y-axis is the prevalence estimates. X-axes are the point-year estimates (1990, 1995, 2000, 2005, 2010, 2017). BMI = Body Mass Index.

**Additional file 2: Table S1.** 95% UI of the age-standardized DALY and Death rates due to high BMI shown in Figure 3. UI=Uncertainty Intervals. DALYs = disability-adjusted life-years; BMI = Body Mass Index.

## Data Availability

All the data used in this article are publicly available online in the official website of Institute of Health Metrics and Evaluation (http://ghdx.healthdata.org/gbd-results-tool).

## Abbreviations

APC: Annualized percent change
BMI: Body mass index
CVD: Cardiovascular disease
DALYs: Disability-adjusted life years
DM: Diabetes mellitus
IBGE: Brazilian Institute of Geography and Statistics
IHME: Institute for Health Metrics and Evaluation
GBD: Global Burden of Disease
GHDx: Global Health Data Exchange
IOTF: International Obesity Task Force
LMIC: Low- and middle-income countries
NCD: Non-communicable diseases
POF: Consumer Expenditure Survey
PAF: Population attributable fraction
ST-GPR: Spatiotemporal Gaussian process regression
TMREL: Theoretical minimum risk exposure level
Vigitel: Telephone Survey Surveillance System for Risk and Protective Factors for Chronic Diseases
UFMG: Universidade Federal de Minas Gerais
UI: Uncertainty interval
YLD: Years lived with disability
YLL: ears of life lost

## References

[1] Abarca-Gómez L, Abdeen ZA, Hamid ZA, Abu-Rmeileh NM, Acosta-Cazares B, Acuin C, et al. Worldwide trends in body-mass index, underweight, overweight, and obesity from 1975 to 2016: a pooled analysis of 2416 population-based measurement studies in 128·9 million children, adolescents, and adults. Lancet. 2017; 10.1016/S0140-6736(17)32129-3. 10.1016/S0140-6736(17)32129-3 PMC5735219 29029897

[2] The GBD 2015 Obesity Collaborators. Health effects of overweight and obesity in 195 countries over 25 years. New England Journal of Medicine. 2017. 10.1056/NEJMoa1614362. 10.1056/NEJMoa1614362 PMC5477817 28604169

[3] Peng W, Berry EM. Global nutrition 1990-2015: A shrinking hungry, and expanding fat world. PLoS One 2018. 10.1371/journal.pone.0194821. eCollection 2018. 10.1371/journal.pone.0194821 PMC5870987 29584768

[4] Singh GM, Danaei G, Farzadfar F, Stevens GA, Woodward M, Wormser D, et al. The age-specific quantitative effects of metabolic risk factors on cardiovascular diseases and diabetes: A pooled analysis. PLoS One. 2013; 10.1371/journal.pone.0065174. 10.1371/journal.pone.0065174 PMC3728292 23935815

[5] Lauby-Secretan B, Scoccianti C, Loomis D, Grosse Y, Bianchini F, Straif K. Body fatness and cancer — viewpoint of the IARC Working Group. N Engl J Med. 2016; https://www.nejm.org/doi/full/10.1056/NEJMsr1606602. 10.1056/NEJMsr1606602 PMC6754861 27557308

[6] Kulkarni K, Karssiens T, Kumar V, Pandit H. Obesity and osteoarthritis. Maturitas. 2016; 10.1016/j.maturitas.2016.04.006. 10.1016/j.maturitas.2016.04.006 27180156

[7] Gurka MJ, Filipp SL, Musani SK, Sims M, DeBoer MD. Use of BMI as the marker of adiposity in a metabolic syndrome severity score: derivation and validation in predicting long-term disease outcomes. Metabolism. 2018. 10.1016/j.metabol.2018.01.015. 10.1016/j.metabol.2018.01.015 PMC5960618 29410278

[8] Malta DC, Andrade SC, Claro RM, Bernal RTI, Monteiro CA. Trends in prevalence of overweight and obesity in adults in 26 Brazilian state capitals and the Federal District from 2006 to 2012. Revista Brasileira de Epidemiologia 2014. 10.1590/1809-4503201400050021. 10.1590/1809-4503201400050021 25054269

[9] Instituto Brasileiro de Geografia e Estatística. Pesquisa Nacional de Saúde, Ciclos de Vida e Medidas Físicas. 2013. http://biblioteca.ibge.gov.br/visualizacao/livros/liv94522.pdf. Acessado 7 dez 2016.

[10] Malta DC, Felisbino-Mendes MS, Machado IE, Passos VMA, Abreu DMX, Ishitani LH, et al. Fatores de risco relacionados à carga global de doença do Brasil e Unidades Federadas, 2015. Revista Brasileira de Epidemiologia. 2017. 10.1590/1980-5497201700050018. 10.1590/1980-5497201700050018 28658385

[11] Lim SS, Vos T, Flaxman AD, Danaei G, Shibuya K, et al. A comparative risk assessment of burden of disease and injury attributable to 67 risk factors and risk factor clusters in 21 regions, 1990-2010: A systematic analysis for the Global Burden of Disease Study 2010. Lancet. 2012; 10.1016/S0140-6736(12)61766-8. 10.1016/S0140-6736(12)61766-8 PMC4156511 23245609

[12] Danaei G, Ding EL, Mozaffarian D, Taylor B, Rehm J, Christopher JLM, Ezzati M. Correction: The preventable causes of death in the United States: comparative risk assessment of dietary, lifestyle, and metabolic risk factors. PLoS Med. 2011; 10.1371/annotation/0ef47acd-9dcc-4296-a897-872d182cde57. 10.1371/journal.pmed.1000058 PMC2667673 19399161

[13] Gakidou E, Afshin A, Abajobir AA, Abate KH, Abbafati C, Abbas KM, et al. Global, regional, and national comparative risk assessment of 84 behavioural, environmental and occupational, and metabolic risks or clusters of risks, 1990–2016: a systematic analysis for the Global Burden of Disease Study 2016. The Lancet. 2017. 10.1016/S0140-6736(17)32366-8. 10.1016/S0140-6736(17)32366-8 PMC5614451 28919119

[14] Forouzanfar MH, Afshin A, Alexander LT, Biryukov S, Brauer M, Cercy K, et al. Global, regional, and national comparative risk assessment of 79 behavioural, environmental and occupational, and metabolic risks or clusters of risks, 1990–2015: a systematic analysis for the Global Burden of Disease Study 2015. Lancet. 2016. 10.1016/S0140-6736(16)31679-8. 10.1016/S0140-6736(16)31679-8 PMC5388856 27733284

[15] Szwarcwald CL, Malta DC, Pereira CA, Vieira MLFP, Conde WL, Souza Júnior PRB, et al. National Health Survey in Brazil: design and methodology of application. Ciênc. saúde coletiva. 2014. 10.1590/1413-81232014192.14072012. 10.1590/1413-81232014192.14072012 24863810

[16] Ricardo, Camila Zancheta, Claro RM. Cost and energy density of diet in Brazil, 2008-2009. Cadernos de Saúde Pública. 2012. 10.1590/S0102-311X2012001400013. 10.1590/s0102-311x2012001400013 23288067

[17] GBD 2017 Risk Factor Collaborators. Global, regional, and national comparative risk assessment of 84 behavioural, environmental and occupational, and metabolic risks or clusters of risks for 195 countries and territories, 1990-2017: a systematic analysis for the Global Burden of Disease Study 2017. Lancet. 2018. 10.1016/S0140-6736(18)32225-6. 10.1016/S0140-6736(18)32225-6 PMC6227755 30496105

[18] Angelantonio ED, Bhupathiraju SN, Wormser D, Gao P, Kaptoge S, Berrington GM, et al. Body-mass index and all-cause mortality: individual-participant-data meta-analysis of 239 prospective studies in four continents. Lancet. 2016. 10.1016/s0140-6736(16)30175-1. 10.1016/S0140-6736(16)30175-1 PMC4995441 27423262

[19] Ezzati, M, Lopez AD, Rodgers A, Vander Hoorn, S, Murray CJL. Selected major risk factors and global and regional burden of disease. Lancet. 2002. 10.1016/S0140-6736(02)11403-6. 10.1016/S0140-6736(02)11403-6 12423980

[20] Pearson-Stuttard J, Zhou B, Kontis V, Bentham J, Gunter MJ, Ezzati M. Worldwide burden of cancer attributable to diabetes and high body-mass index: a comparative risk assessment. Lancet Diabetes Endocrinol. 2018; 10.1016/S2213-8587(18)30150-5. 10.1016/S2213-8587(17)30366-2 PMC5805864 29195904

[21] Das Gupta P. Standardization and decomposition of rates: a user’s manual. Washington D.C.: U.S. Bureau of the Census; 1993. https://www2.census.gov/library/publications/1993/demographics/p23-186.pdf.

[22] Supplemental appendix 8. GBD 2015 Risk Factors Collaborators. Supplementary appendix. The Lancet. 2016. http://www.thelancet.com/cms/attachment/2067427612/2067048266/mmc1.pdf. Acessed 10 Jan 2017..

[23] Araújo FG, Velasquez-Melendez G, Felisbino-Mendes MS. Prevalence trends of overweight, obesity, diabetes and hypertension among Brazilian women of reproductive age based on sociodemographic characteristics. Health Care for Women International. 2019; 10.1080/07399332.2019.1570516. 10.1080/07399332.2019.1570516 30986134

[24] Maria Aiello A, Marques de Mello L, Souza Nunes M, Soares da Silva A, Nunes A. Prevalence of obesity in children and adolescents in Brazil: a meta-analysis of cross-sectional studies. Current Pediatric Reviews. 2015. 10.2174/1573396311666150501003250. 10.2174/1573396311666150501003250 25938377

[25] Flores-Ortiz R, Malta DC, Velasquez-Melendez G. Adult body weight trends in 27 urban populations of Brazil from 2006 to 2016: a population-based study. PLoS One. 2019; 10.1371/journal.pone.0213254. 10.1371/journal.pone.0213254 PMC6402686 30840675

[26] Flores LS, Gaya AR, Petersen RD, Gaya A. Trends of underweight, overweight, and obesity in Brazilian children and adolescents. J Pediatr (Rio J). 2013. 10.1016/j.jped.2013.02.021. 10.1016/j.jped.2013.02.021 23850108

[27] IBGE (2010). Pesquisa de Orçamentos Familiares 2008-2009: despesas, rendimentos e condições de vida.

[28] Ng M, Fleming T, Robinson M, Thomson B, Graetz N, Margono C, et al. Global, regional, and national prevalence of overweight and obesity in children and adults during 1980–2013: a systematic analysis for the Global Burden of Disease Study 2013. Lancet. 2014. 10.1016/S0140-6736(14)60460-8. 10.1016/S0140-6736(14)60460-8 PMC4624264 24880830

[29] Swinburn BA, Sacks G, Hall KD, McPherson K, Finegood DT, Moodie ML, et al. The global obesity pandemic: Shaped by global drivers and local environments. Lancet. 2011; 10.1016/S0140-6736(11)60813-1. 10.1016/S0140-6736(11)60813-1 21872749

[30] Pessoa MC, Mendes LL, Gomes CS, Martins PA, Velasquez-Melendez G. Food environment and fruit and vegetable intake in a urban population: a multilevel analysis. BMC Public Health. 2015. 10.1186/s12889-015-2277-1. 10.1186/s12889-015-2277-1 PMC4595198 26437719

[31] Jaime PC, Duran AC, Sarti FM, Lock K. Investigating environmental determinants of diet, physical activity, and overweight among adults in Sao Paulo. Brazil Journal of Urban Health. 2011; 10.1007/s11524-010-9537-2. 10.1007/s11524-010-9537-2 PMC3126927 21327549

[32] Marinho F, Azeredo PVM, Malta DC, França EB, Abreu DMX, Araújo VEM, et al. Burden of disease in Brazil, 1990–2016: a systematic subnational analysis for the Global Burden of Disease Study 2016. Lancet. 2018; 10.1016/S0140-6736(18)31221-2. 10.1016/S0140-6736(18)31221-2 PMC6123514 30037735

[33] Mokdad AH. Burden of cardiovascular diseases in the Eastern Mediterranean Region, 1990–2015: findings from the Global Burden of Disease 2015 study. Int J Public Health. 2017. 10.1007/s00038-017-1012-3. 10.1007/s00038-017-1012-3 PMC5973984 28776245

[34] Agrawal S, Agrawal PK (2016). Association between body mass index and prevalence of multimorbidity in low-and middle-income countries: a cross-sectional study. Int J Med Public Health.

[35] Peters SAE, Woodward M, Jha V, Kennedy S, and Norton R. Women’s health: a new global agenda. BMJ Glob Health. Published online. 2016. 10.1136/bmjgh-2016-000080. 10.1136/bmjgh-2016-000080 PMC5321350 28588958

[36] Al-Goblan AS, Al-Alfi AM, Khan MZ. Mechanism linking diabetes mellitus and obesity. Diabetes Metab Syndr Obes. Published online. 2014. 10.2147/DMSO.S67400. 10.2147/DMSO.S67400 PMC4259868 25506234

[37] Lu Y, Hajifathalian K, Ezzati M, Woodward, Rimm EB, Danaei G. Metabolic mediators of the effects of body-mass index, overweight, and obesity on coronary heart disease and stroke: a pooled analysis of 97 prospective cohorts with 1.8 million participants. Lancet. 2014. 10.1016/s0140-6736(13)61836-x. 10.1016/S0140-6736(13)61836-X PMC3959199 24269108

[38] Gaal LFV, Mertens IL, Block CE (2006). Mechanisms linking obesity with cardiovascular disease. Nature..

[39] O'Brien PD, Hinder LM, Callaghan BC, Feldman EL (2017). Neurological consequences of obesity. The Lancet Neurology.

[40] Brazil. Health Surveillance Secretariat. Health Situation Analysis Department. Strategic action plan to tackle noncommunicable diseases (NCD) in Brazil 2011-2022 / Ministry of Health. Health Surveillance Secretariat. Ministry of Health. 2011. https://www.iccp-portal.org/system/files/plans/BRA_B3_Plano%20DCNT%20-%20ingl%C3%AAs.pdf. Accessed 28 Oct 2018.

[41] Malta DC, Silva Júnior JB. Policies to promote physical activity in Brazil. The Lancet. 2012. https://www.thelancet.com/journals/lancet/article/PIIS0140-6736(12)61041-1/fulltext. 10.1016/S0140-6736(12)61041-1 22818935

[42] Jaime PC, Silva AC, Gentil PC, Claro RM, Monteiro CA. Brazilian obesity prevention and control initiatives. Obes Rev. 2013; 10.1111/obr.12101. 10.1111/obr.12101 24102701

[43] Duncan BB, França EB, Passos VMA, Cousin E, Ishitani LH, Malta DC, et al. The burden of diabetes and hyperglycemia in Brazil and its states: findings from the Global Burden of Disease Study 2015. Revista Brasileira de Epidemiologia. 2017. 10.1590/1980-5497201700050008. 10.1590/1980-5497201700050008 28658375

[44] Gortmaker SL, Wang YC, Long MW, Giles CM, Ward ZJ, Barrett JL, et al. Three interventions that reduce childhood obesity are projected to save more than they cost to implement. Health Aff. 2015; 10.1377/hlthaff.2015.0631. 10.1377/hlthaff.2015.0631 PMC9627551 26526252

[45] Asif M. The prevention and control the type-2 diabetes by changing lifestyle and dietary pattern. J Educ Health Promot. 2014;3:1. Published online. 2014. 10.4103/2277-9531.127541. 10.4103/2277-9531.127541 PMC3977406 24741641

[46] Douketis JD, Macie C, Thabane L, Williamson DF. Systematic review of long-term weight loss studies in obese adults: Clinical significance and applicability to clinical practice. Int J Obes. 2005; 10.1038/sj.ijo.0802982. 10.1038/sj.ijo.0802982 15997250

[47] Moreira NF, Luz VG, Moreira CC, Pereira RA, Sichieri R, Ferreira MG, et al. Self-reported weight and height are valid measures to determine weight status: results from the Brazilian National Health Survey (PNS 2013). Cadernos de Saúde Pública. 2018;34(5):e00063917. Epub May 10, 2018. 10.1590/0102-311x00063917. 10.1590/0102-311X00063917 29768583

